# Observations on Wounds of the Heart, and Their Relations to Forensic Medicine

**Published:** 1855-07

**Authors:** 


### OBSERVATIONS ON WOUNDS OF THE HEART, AND THEIR RELATIONS TO FORENSIC MEDICINE. By S. Purple, M. D.

In the small space of thirty-two pages Dr. Purple has furnished a map of statistics of injuries of the heart, with a table of forty-two cases, which will interest every physician. From the cases which he has collated he has drawn the following conclusions:—That wounds of the heart are not in general immediately fatal; that recovery is possible, and provided a careful and judicous treatment is adopted is almost probable; that the presence of a leaden ball in the walls of the ventricle does not preclude either recovery or the continuance of life for a number of years; that incised wounds of the heart may heal by adhesion; that the diagnosis of wounds of the heart is uncertain, and the prognosis unfavorable; that the treatment is that adapted to wounds of the chest in general; that the right ventricle is the portion of the heart most frequently injured; that the average duration of life is greater if the left ventricle be the seat of the injury. This proposition, it will be remarked, is opposed to the opinion of almost all writers on the subject. D. W. Y.

